# Chilblains and Chapped Hands

**Published:** 1871-04

**Authors:** 


### CHILBLAINS AND CHAPPED HANDS.

The returning cold, damp weather brings in its train the seasonable series of complaints, such as chilblains, chapped hands and lips, etc. These appear to be most prevalent just now, amongst those exposed to the inclemency of changeable weather, who possess a fair complexion, delicate skin, and other constitutional predispositions. To those especially liable to these tiresome and painful affections, we recommend as a preventive wearing kid skin gloves lined with wool, which not only keep out the cold, but absorb any moisture that may be upon the hands; and to rub over the hands before washing a small quantity of glycerine, which should be allowed to dry or become absorbed to a partial extent. When chilblains manifest themselves, the best remedy not only for preventing their ulcerating, but overcoming the tingling, itching pain and stimulating the circulation of the part to healthy action, is the liniment of belladonna (two drachms), the liniment of aconite (one drachm), carbolic acid (ten drops), collodion flexile (one ounce) painted with a camel's-hair pencil over their surface. When the chilblains vesicate, ulcerate, or slough, it is better to omit the aconite, and apply the other components of the liniment without it. The collodion flexile forms a coating or protecting film, which excludes the air, whilst the sedative liniments allay the irritation generally of no trivial nature. For chapped hands we advise the free use of glycerine and good olive oil in the proportion of two parts of the former to four of the latter; after this has been well rubbed into the hands and allowed to remain for a little time, and the hands subsequently washed with Castile soap and tepid water, we commend the belladonna and collidon flexile to be painted, and the protective film allowed to remain permanently. These complaints not unfrequently invade persons of languid circulation and relaxed habit, who should be put on a nutritious regimen and treated with ferruginous tonics. Obstinate cases are occasionally met with which no local application will remedy, until some disordered state of the system is removed, or the general condition of the patient's health improved. Chapped lips are also benefited by the stimulating form of application we advocate, but the aconite must not be allowed to get on the lips, or a disagreeable tingling results.—*London Chemist and Druggist.*

